# The cuproptosis-associated 13 gene signature as a robust predictor for outcome and response to immune- and targeted-therapies in clear cell renal cell carcinoma

**DOI:** 10.3389/fimmu.2022.971142

**Published:** 2022-09-05

**Authors:** Huiyang Yuan, Xin Qin, Jing Wang, Qingya Yang, Yidong Fan, Dawei Xu

**Affiliations:** ^1^ Department of Urology, Qilu Hospital of Shandong University, Jinan, China; ^2^ Department of Urologic Oncology, The First Affiliated Hospital of University of Science and Technology of China (USTC), Division of Life Sciences and Medicine, University of Science and Technology of China, Hefei, China; ^3^ Department of Medicine, Division of Hematology, Bioclinicum and Center for Molecular Medicine, Karolinska Institute and Karolinska University Hospital Solna, Stockholm, Sweden

**Keywords:** ccRCC, cuproptosis, immunotherapy, immune checkpoint inhibitors, prognosis, targeted therapy

## Abstract

Cuproptosis, the newly identified form of regulatory cell death (RCD), results from mitochondrial proteotoxic stress mediated by copper and FDX1. Little is known about significances of cuproptosis in oncogenesis. Here we determined clinical implications of cuproptosis in clear cell renal cell carcinoma (ccRCC). Based on the correlation and survival analyses of cuproptosis-correlated genes in TCGA ccRCC cohort, we constructed a cuproptosis-associated 13 gene signature (CuAGS-13) score system. In both TCGA training and two validation cohorts, when patients were categorized into high- and low-risk groups according to a median score as the cutoff, the CuAGS-13 high-risk group was significantly associated with shorter overall survival (OS) and/or progression-free survival (PFS) independently (*P*<0.001 for all). The CuAGS-13 score assessment could also predict recurrence and recurrence-free survival of patients at stage I – III with a high accuracy, which outperformed the ccAccB/ClearCode34 model, a well-established molecular predictor for ccRCC prognosis. Moreover, patients treated with immune checkpoint inhibitors (ICIs) acquired complete/partial remissions up to 3-time higher coupled with significantly longer PFS in the CuAGS-13 low- than high-risk groups in both training and validation cohorts of ccRCCs (7.2 – 14.1 *vs.* 2.1 – 3.0 months, *P*<0.001). The combination of ICI with anti-angiogenic agent Bevacizumab doubled remission rates in CuAGS-13 high-risk patients while did not improve the efficacy in the low-risk group. Further analyses showed a positive correlation between CuAGS-13 and TIDE scores. We also observed that the CuAGS-13 score assessment accurately predicted patient response to Sunitinib, and higher remission rates in the low-risk group led to longer PFS (Low- *vs.* high-risk, 13.9 *vs.* 5.8 months, *P* = 5.0e-12). Taken together, the CuAGS-13 score assessment serves as a robust predictor for survival, recurrence, and response to ICIs, ICI plus anti-angiogenic drugs and Sunitinib in ccRCC patients, which significantly improves patient stratifications for precision medicine of ccRCC.

## Introduction

Clear cell renal cell carcinoma (ccRCC), derived from the epithelial cells in the nephron, is the predominant subtype of renal cell carcinoma (RCC) (up to 80% of all RCCs), and characterized by the inactivation of the *von Hippel Lindau (VHL)* gene and subsequent dysregulation of hypoxia-inducible factor (HIF)-responsive genes (1–4). ccRCC incidence has increased over the past decades worldwide (3), while fortunately, most patients are diagnosed at early stages with localized disease, and thus successfully resected (2). However, approximately 30% of these patients will undergo recurrence post-operation (2). Traditionally, patient clinicopathological features are applied to evaluate recurrence risk and to predict prognosis (5). More recently, efforts have been made to identify molecular biomarkers for reliable outcome prediction of ccRCC (5). Towards this purpose, several studies developed multigene expression signatures, and these signatures, either alone or together with the traditional stratification system, were shown to improve ccRCC prognostication (5–11). Despite so, molecular and clinicopathological parameters are still far from accurately predicting patient outcomes. It is thus demanding tasks to further develop new biomarkers or molecular tools for ccRCC prognosis and personalized interventions.

ccRCC is intrinsically insensitive to chemotherapy, and therefore, other treatment strategies have been applied (12). For instance, interleukin 2 (IL2), as an immunotherapeutic agent, has been widely used for metastatic ccRCC (mccRCC) since decades ago, which achieved complete and durable responses in a fraction of patients (13, 14). However, severe side-effects significantly restricted the application of IL2 treatment (15). More recently, boosting anti-cancer immune response using immune checkpoint inhibitors (ICIs) have revolutionized the cancer therapy (15, 16). By targeting immune checkpoint proteins PD-1/PDL-1 and/or CTLA4, the ICI strategy shows clinical benefits in various cancer types. Similarly, this approach has been successful in the treatment of localized ccRCC as adjuvant therapy after nephrectomy and mccRCC. However, response rates for ccRCC are in general less than 50% (15, 17). The combined treatment of ICIs with targeted therapeutic drugs such as Bevacizumab may improve efficacy (18–20). ccRCC exhibits unique immunological features, and high CD8 T infiltration correlates with poor prognosis, which contrasts with favorable outcomes observed in other cancer types (16, 21). In addition, tumor mutation burden (TMB) predicts ICI response in many solid tumors, but not in ccRCC (22, 23). PBRM1 mutations and expression of human endogenous retroviruses (HERVs) were shown to be associated with response in ccRCC by some studies but could not be validated in other reports (22, 24–27). More recently, other biomarkers have been developed to predict patient response to ICIs (21, 28). Thus, identifying reliable predictors for ICI response in ccRCC remains unmet demands. It is also poorly defined which patients will benefit more from the combined therapy of ICIs with Bevacizumab, which calls for further investigations.

In addition, Sunitinib, an inhibitor of multiple tyrosine-kinase receptors, was approved by FDA for the first line treatment of ccRCC in 2006 (29). Most patients benefit from the treatment with longer progression-free survival (PFS), but approximately 1/3 of ccRCCs exhibit intrinsic resistance to Sunitinib (12). Distinguishing Sunitinib responders from non-responders is clinically important.

One of the cancer hallmarks is an increased capacity for survival (30). Evading apoptosis is the well-defined mechanism for cancer cells to evade death fate (30). There exist other forms of regulated cell death (RCD), such as ferroptosis, paraptosis and pyroptosis, and they similarly play a part in modulating cancer cell survival (31). More recently, a copper-dependent cell death, so-called cuproptosis, was identified by Tsvetkov et al. (32). Mechanistically, the reductase FDX1 and copper induce the lipoylation and aggregation of mitochondrial enzymes responsible for the tricarboxylic acid (TCA) cycle, and promote Fe-S cluster protein degradation, thereby leading to proteotoxic stress and cell death (32). It is currently unclear whether cuproptosis contributes to the ccRCC pathogenesis and has any clinical implications in ccRCC managements. The present study is designed to address these issues. By analyzing TCGA and other datasets, we identified the cuproptosis-associated 13 gene signature (CuAGS-13) as a predictor for patient survival, recurrence and response to ICI, Bevacizumab and Sunitinib treatments in ccRCC.

## Materials and methods

### Data collection and processing of ccRCC tumors

The TCGA cohort of ccRCCs included 525 tumor samples with survival information available and 72 nontumorous adjacent renal tissues (11). Transcriptome, mutation, copy number variations (CNAs) and clinical-pathological data were downloaded from https://gdc.cancer.gov/. One hundred and one patients with ccRCC were in the E-MTAB-1980 cohort (33), and RNA array and clinical information were downloaded from http://www.ebi.ac.uk. The ICGC-RECA-EU cohort included 91 ccRCC patients and their clinical and RNA sequencing data were downloaded from https://dcc.icgc.org/. For RNA sequencing data, mRNA abundances were expressed as Transcripts Per Million (TPM). Microarray data of patient-derived xenografts *(*PDX*)* models in GSE64052 were downloaded from the Gene Expression Omnibus database (https://www.ncbi.nlm.nih.gov/geo/). For array results (determined by 4×44K v2 microarray kit) from the E-MTAB-1980 cohort and GSE64052, probe-set values were used to quantify mRNA levels. ccRCC patients receiving ICIs, ICIs plus Bevacizumab, and Sunitinib treatments were contained in IMmotion150 (34, 35), CheckMate025 (23, 24) and IMmotion151 trials (18, 36). No ethics approval is required for the present study.

### Identification of cuproptosis-associated genes using weighted gene co-expression network analysis

For 525 tumors and 72 adjacent non-cancerous renal tissues in the TCGA ccRCC cohort, the single sample gene set enrichment (ssGSEA) analysis was carried out to calculate the cuproptosis ssGSEA score in each sample according to expression levels of 10 cuproptosis genes (*FDX1*, *LIAS*, *LIPT1*, *DLD*, *DLAT*, *PDHA1*, *PDHB*, *MTF1*, *GLS* and *CDKN2A*). The enrichment statistic (ES) value in each sample (ssGSEA score) was calculated using GSVA package based on standardized mRNA levels [log2(TPM+1)] of each sample. WGCNA analyses were then performed to establish a co-expression network based on the cuproptosis ssGSEA score (**Figure S1**). Towards this end, hierarchical clustering by average link first detected outliner samples for exclusion (**Figure S1A**) and the Pearson’s correlation matrices were then applied for all pair-wise genes followed by the construction of a weighted adjacency matrix. The soft-thresholding parameter or β value, which highlights strong correlations while penalizes weak correlations between genes, was set at 6 (scale free R^2^ = 0.80) for a scale-free net-work based on the scale independence and mean connectivity (**Figure S1B**). The generated adjacency matrix was further transformed into the topological overlap matrix (TOM). All genes were categorized into co-expression modules according to TOM-based dissimilarity using an average linkage hierarchical clustering method. The first principal component of expression matrix is set as module eigengenes (**Figure S1C**). A total of 27 modules were finally identified (**Figure S1C**). Among these modules, brown and magenta ones were highly correlated with the cuproptosis score (**Figure 2A**). In addition, tumor and immune scores were integrated into the analysis of the relationship between cuproptosis score and tumor/immune scores (**Figure 2A**).

### Construction of the CuAGS-13 risk score

Using the threshold for module membership correlation >0.5 and gene significance Cor >0.2, we acquired a total of 872 genes, among which 771 genes (in brown modules) correlated with while 101 genes (in magenta module) anti-correlated with the cuproptosis score. The impact of these 872 gene levels on progression-free survival (PFS) was evaluated using univariate COX regression and K-M analyses and 315 genes were selected for further analysis by the least absolute shrinkage and selector operation (LASSO) regression. Thirteen genes were finally acquired as the cuproptosis-associated gene signature or CuAGS-13 after verification by the Cox proportional-hazards model. We calculated CuAGS-13 score in each sample based on the following formula:

Score = Σ βi × RNAi, where βi is the coefficient of the i-th gene in multivariable Cox regression analysis, and RNAi is RNA expression level of gene i. Patients were divided into the high- and the low-risk groups using the median score as a cut-off. Differences in survival (OS, PFS and RFS), recurrence, and response to ICIs or Sunitinib between the high- and low-risk groups were analyzed using packages of the R software. The accuracy of the prediction is evaluated using the ROC curve. For comparison with the ccA/ccB/ClearCode34 model, the classification of the TCGA cohort was directly from published data by Brook et al. (7) and Buttner et al. (8).

Expression differences in CuAGS-13-containing 13 genes were compared between ccRCC tumors and non-tumorous adjacent renal tissues in the TCGA cohort. For RNA expression, log2(TPM+1) based on RNA sequencing data was form https://gdc.cancer.gov/ as stated above. Protein expression data was obtained from Clinical Proteomic Tumor Analysis Consortium (http://ualcan.path.uab.edu/index.html).

### Development of a predictive nomogram for survival and recurrence

Cox regression analysis was performed to determine the impact of the CuAGS-13 score and clinical variables on survival and recurrence. Thereafter, based on multivariate Cox regression analysis results, we constructed a predictive nomogram that included CuAGS-13 score, age, grade and stage to predict 1-, 3-, and 5-year survival (OS, PFS and/or RFS) and recurrence. Predicted survival of the nomogram against observed ones was plotted using the calibration curve. All nomograms and assessments of their predicative powers were made using R package regplot.

### TIDE score analysis for response to ICIs

TIDE score is calculated based on myeloid-derived suppressor cell (MDSC), macrophage M2, T cell Dysfunction and Exclusion (37). TCGA ccRCC TIDE score was directly downloaded from http://tide.dfci.harvard.edu/. TIDE score for ccRCC cohort treated with Nivolumab was calculated online at http://tide.dfci.harvard.edu/. mRNA expression was standardized by using the all sample average expression as the normalization control prior to TIDE score analysis.

### Gene set enrichment analysis

GSEA for KEGG (GSEA-KEGG) and Hallmark (GSEA-Hallmark) pathways (version 4.2.1 www.broadinstitute.org/gsea) was carried out to determine CuAGS-13 score-related signaling enrichments. Adjusted *P* < 0.05 and FDR <0.25 were defined as the activation or inhibition of signaling pathways.

### Statistical analysis

All statistical analyses were carried out using R package version 4.0.5. Wilcox and K-W sum tests were used for analysis of differences between two groups and among multi groups, respectively. Spearman’s Rank-Order Correlation coefficient was applied to determine correlation coefficients r between two variables. Survival analyses were made using log-rank test. The Survival and Survminer packages were employed to draw Kaplan–Meier survival curves for visualization of OS, PFS and RFS. Univariate and multivariate Cox regression analyses were used to determine the effect (HR and 95% CI) of various quantitative predictor variables on OS, PFS or RFS. Time-dependent ROCs and AUCs were made using Rpackage timeROC. *P* < 0.05 were considered as statistically significant.

## Results

### Construction of a cuproptosis-associated gene signature in the TCGA cohort of ccRCC

**Figure 1:**
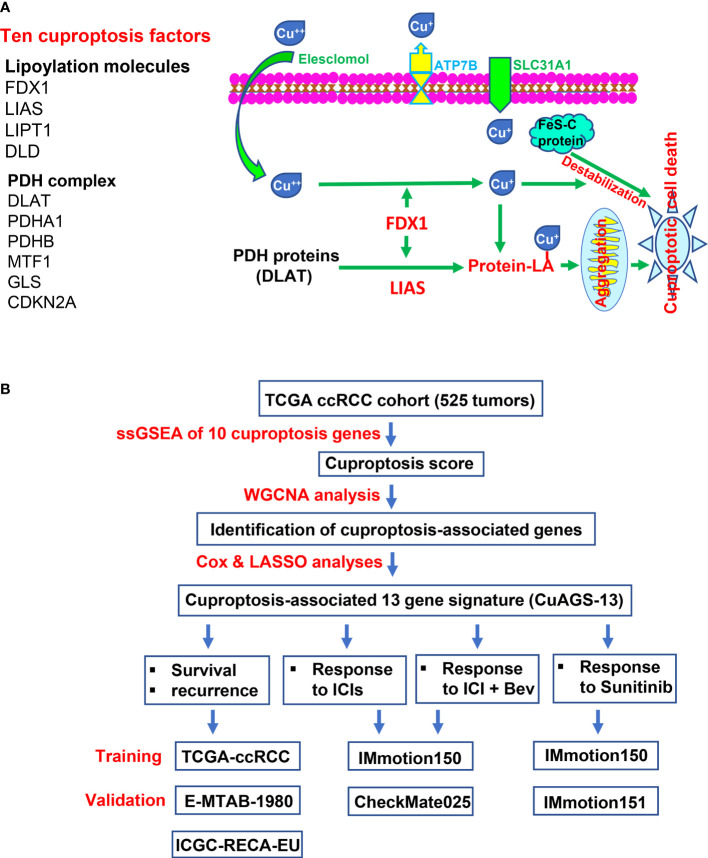
The Cuproptosis pathway and study workflow. **(A)** Left panel: Ten factors involved in cuproptosis. Right panel: The cuproptosis signaling pathway. Extracellular copper Cu^++^ enters cells by binding to copper chelators and elesclomol serves as the most efficient Cu^++^ transporter. The reductase FDX1 reduces Cu^++^ to Cu^+^, a more toxic form, while lipoyl synthase (LIAS) catalyzes lipoylation of the pyruvate dehydrogenase (PDH) complex proteins including dihydrolipoamide S-acetyltransferase (DLAT) and others. Cu^+^ and lipoylation promote the protein aggregation. DLAT is one of the key enzymes participating in the *tricarboxylic acid* cycle, and its aggregation results in mitochondrial proteotoxic stress and subsequent cuproptotic cell death. Moreover, FDX1 and Cu^+^ induce the destabilization of Fe–S cluster proteins, further facilitating cuproptosis. Additionally, SLC31A1 and ATP7B function as the Cu^+^ importer and exporter, respectively, and regulate cuproptosis by controlling intracellular Cu^+^ concentrations. **(B)** The schematic workflow of the present study.

**Figure 2:**
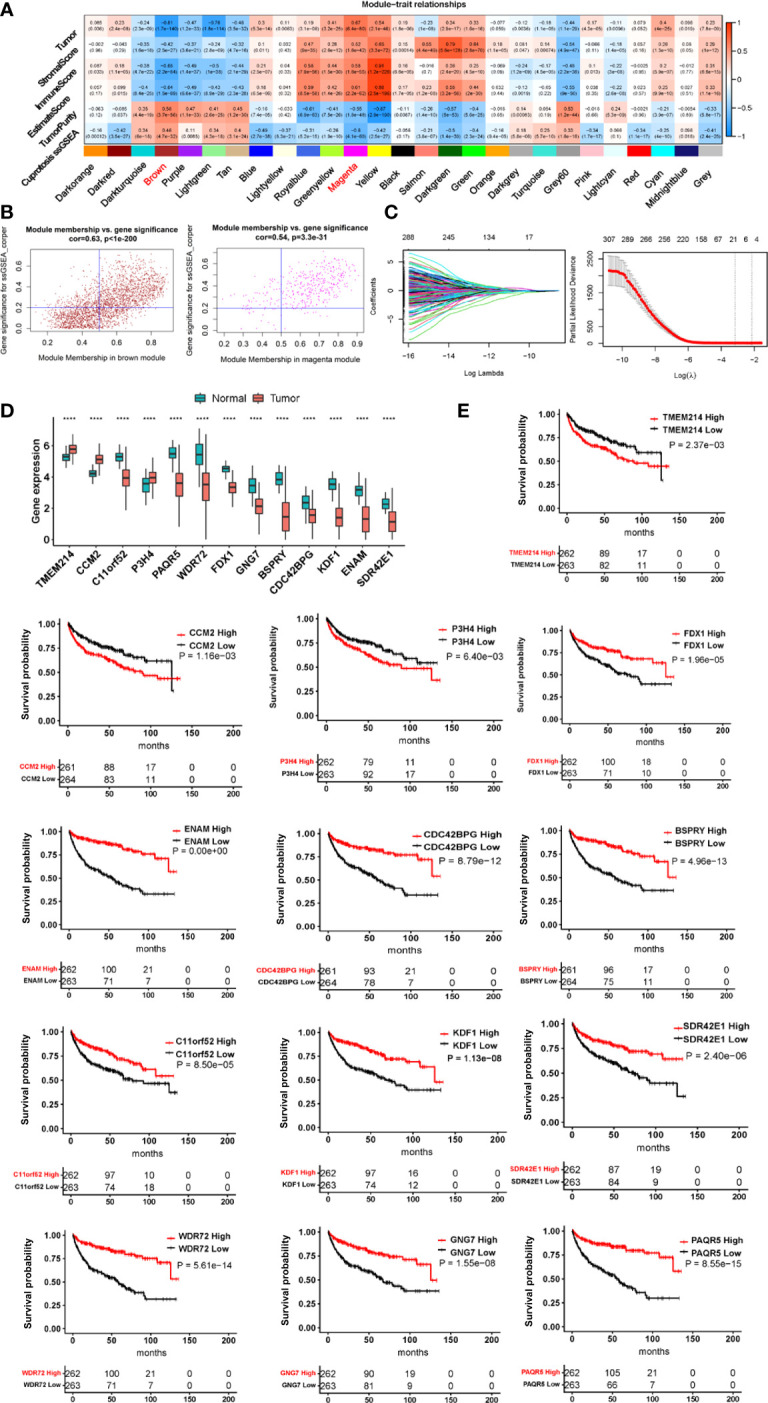
The construction of the cuproptosis-associated 13 gene signature (CuAGS-13) for ccRCC prognosis. **(A)** Left panel: Gene modules correlated with cuproptosis factors as determined using Weighted gene co-expression network analysis (WGCNA) and Pearson’s co-efficiency analysis. **(B)** Scatter plot of module eigengenes in the MEBROWN (left) and MEMANGE (right) modules from **(A)**. The genes in the upper right are selected for further analyses. **(C)** Construction of the cuproptosis-associated 13 gene signature (CuAGS-13) for progression-free survival (PFS) prediction in ccRCC. Top panel: LASSO coefficient profiles of the CuAGS associated with PFS. Bottom panel: Plots of the cross-validation error rates. Each red dot represents a lambda value with its error bar (the confidence interval for the cross-validated error rate). The analysis identified 13 cuproptosis-associated genes most relevant to PFS. **(D)** Differences in the CuAGS-13 expression between ccRCC tumors and their non-tumorous adjacent renal tissues in the TCGA cohort. **(E)** Kaplan–Meier survival analysis showing the impact of each gene contained in CuAGS-13 on PFS in the TCGA ccRCC cohort. Patients are divided into high and low groups based on the expression of each gene in tumors using a median value as the cutoff. ****p < 0.0001.

Ten factors, which include FDX1, LIAS, LIPT1, DLD, DLAT, PDHA1, PDHB, MTF1, GLS, and CDKN2A, have been identified to participate in the cuproptosis process (32) (**Figure 1A**). Among these factors, FDX1 functions as a key player to drive cuproptosis by reducing Cu^++^ to Cu^+^ (32) (**Figure 1A**). Because it is currently unclear which roles cuproptosis has in ccRCC pathogenesis, we first sought to determine whether these 10 molecules were associated with patient survival but failed to establish a satisfactory model in the TCGA cohort of ccRCC (**Supplementary figures 2** and **3**). We then made ssGSEA analysis to calculate the cuproptosis score in each sample based on the expression of 10 genes above, followed by the weighted gene co-expression network analysis (WGCNA) to look for cuproptosis-correlated genes (**Figure 1B**). By doing so, we identified that the cuproptosis score was (i) significantly correlated with 771 while anti-correlated with 101 genes; (ii) negatively associated with oncogenesis, indicating a tumor suppressive role of cuproptosis; and (iii) significantly correlated with immuneEstimate scores (**Figure 2A**). COX and LASSO regression analyses were then carried out to assess the impact of these 872 genes on patient progression-free survival (PFS) (**Figures 2A–C**). We finally acquired 13 genes as the cuproptosis-associated 13 gene signature, which we named as CuAGS-13. These 13 genes include TMEM214, CCM2, P3H4, FDX1, CDC42BPG, C11orf52, GNG7, PAQR5, ENAM, WDR72, SDR42E1, BSPRY and KDF1. The cuproptosis score was correlated negatively with the expression of TMEM214, CCM2 and P3H4, while positively with the rest of them. TMEM214, CCM2 and P3H4 expression was significantly higher in tumors than in their normal counterpart tissues (**Figure 2D**). In contrast, the expression of the rest 10 genes was dramatically downregulated in tumors (**Figure 2D**). Further CPTAC analyses of their protein expression (9 of 13 protein expression data available) showed that differences in protein levels between normal and tumors were largely similar to RNA expression trends (**Figure S4**). Each of these 13 factors was significantly associated with PFS when patients were divided into high and low categories using a median value as the cutoff (**Figure 2E**). In addition, the CuAGS-13 score was significantly associated with multi clinical-pathological variables including age, gender, grade, stage, metastasis and white cells in the TCGA ccRCC cohort (**Table S1**).

### The CuAGS-13 score for survival prediction in ccRCC

**Figure 3:**
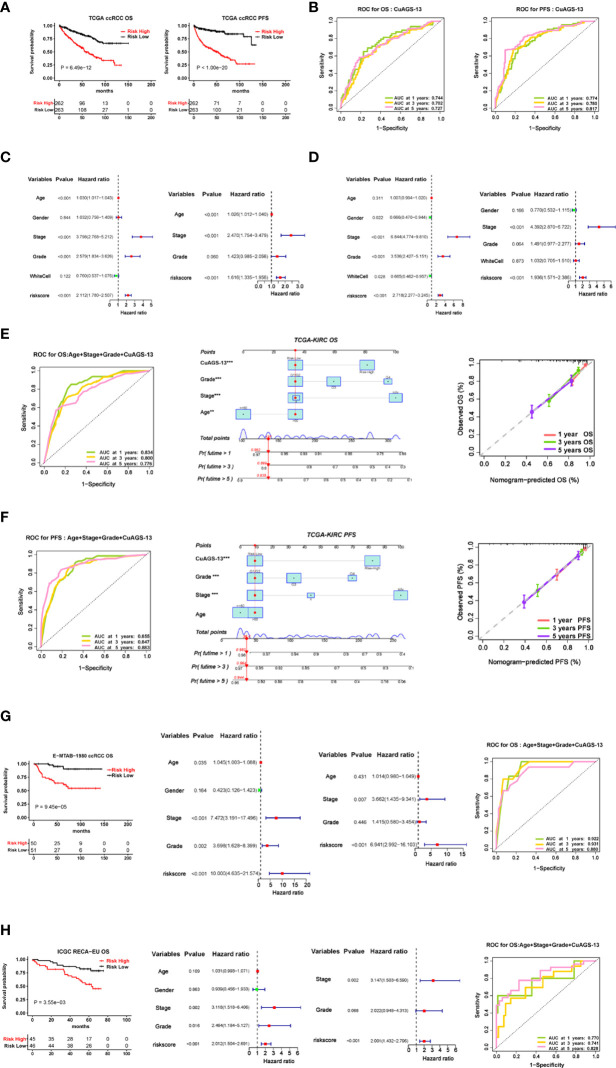
The cuproptosis-associated 13 gene signature (CuAGS-13) model for ccRCC survival prediction. **(A)** Kaplan–Meier survival analysis showing the significant association of the CuGAS-13 score with OS and PFS in the TCGA ccRCC cohort. Patients were classified into high- and low-risk groups based on the CuGAS-13 score using a median value as the cutoff. **(B)** The ROC curve showing a high accuracy in predicting 1-, 3- and 5-year OS and PFS using the CuGAS-13 model. **(C)** and **(D)** Univariate and multivariate Cox regression analyses of OS and PFS in ccRCC, respectively. **(E)** and **(F)** The nomogram composed of CuAGS-13 model, age, grade and stage for predicting 1-, 3- and 5-year OS and PFS, respectively. **(G)** The validation of the CuGAS-13 model for the prediction of OS in the EMBA-1980 cohort of ccRCC. **(H)** The validation of the CuGAS-13 model for the prediction of OS in the ICGC-RECA-EU cohort of ccRCC.

We then sought to determine impacts of the CuAGS-13 score on OS and PFS in 525 ccRCC patients from the TCGA dataset as a training cohort (**Table S1**). According to the CuAGS-13 score in ccRCC tumors, patients were categorized into high- and low-risk groups using the median score value as a cut-off (> and ≤ median score, respectively). A Kaplan-Meier analysis revealed that patients in the high-risk group had significantly shorter OS and PFS (*P*<1e-11 and 1e- 20, respectively) (**Figure 3A**). The risk score exhibited a high accuracy in predicting 1-, 3- and 5-year survival, as assessed by a time-dependent Receiver Operator Characteristic (ROC) curve (**Figure 3B**). Univariate COX regression survival analyses were further performed by including patient age, gender, stage, grade, and white cells together with the CuAGS-13 model. As shown in **Figures 3C, D**, stage, grade and CuAGS-13 score (high-risk) were all significantly associated with shorter OS and PFS, while White cells were associated with longer PFS without affecting OS, and female patients had longer PFS. Age was associated with shorter OS but not PFS. Multivariate analyses revealed that stage, grade and CuAGS-13 score (high-risk) were all independent prognostic factors for shorter OS and PFS, while age remained as a variable associated with shorter OS (**Figure 3C**). Based the results above, we established a prognostic nomogram composed of CuAGS-13 score, age, stage, and grade, which showed a highly accurate estimation of survival possibilities at 1, 3 and 5 years (**Figures 3E, F**).

To confirm the findings in the TCGA ccRCC, we further assessed the effect of the CuAGS-13 score on survival of ccRCC patients from two other databases as validation cohorts. For the E-MTAB-1980 cohort of 101 patients (33), OS data were available, and their clinic-pathological characteristics were listed in **Table S2**. The CuAGS-13 score high-risk group had significantly shorter OS (*P* = 9.45e-10^5^) and served as an independent prognostic factor as revealed by the multivariate Cox regression analysis (**Figure 3G**). The ROC curve further showed a robust power in predicting 1-, 3- and 5-year survival when the CuAGS-13 model was combined with age, grade and stage (**Figure 3G**). The ICGC-RECA-EU cohort included 91 ccRCC patients (**Table S3**) (https://dcc.icgc.org/) and our analysis results were very similar to those observed in E-MTAB-1980 cohort (**Figure 3H**). In those 91 patients, adjacent normal renal tissues from 45 were also analyzed for their expression profile, and the comparison in 13 gene expression between tumors and normal tissues showed largely same patterns as seen in the TCGA cohort except CDC42BPG (**Figure S5**).

### The recurrence prediction of ccRCC patients by the CuAGS-13 model

**Figure 4:**
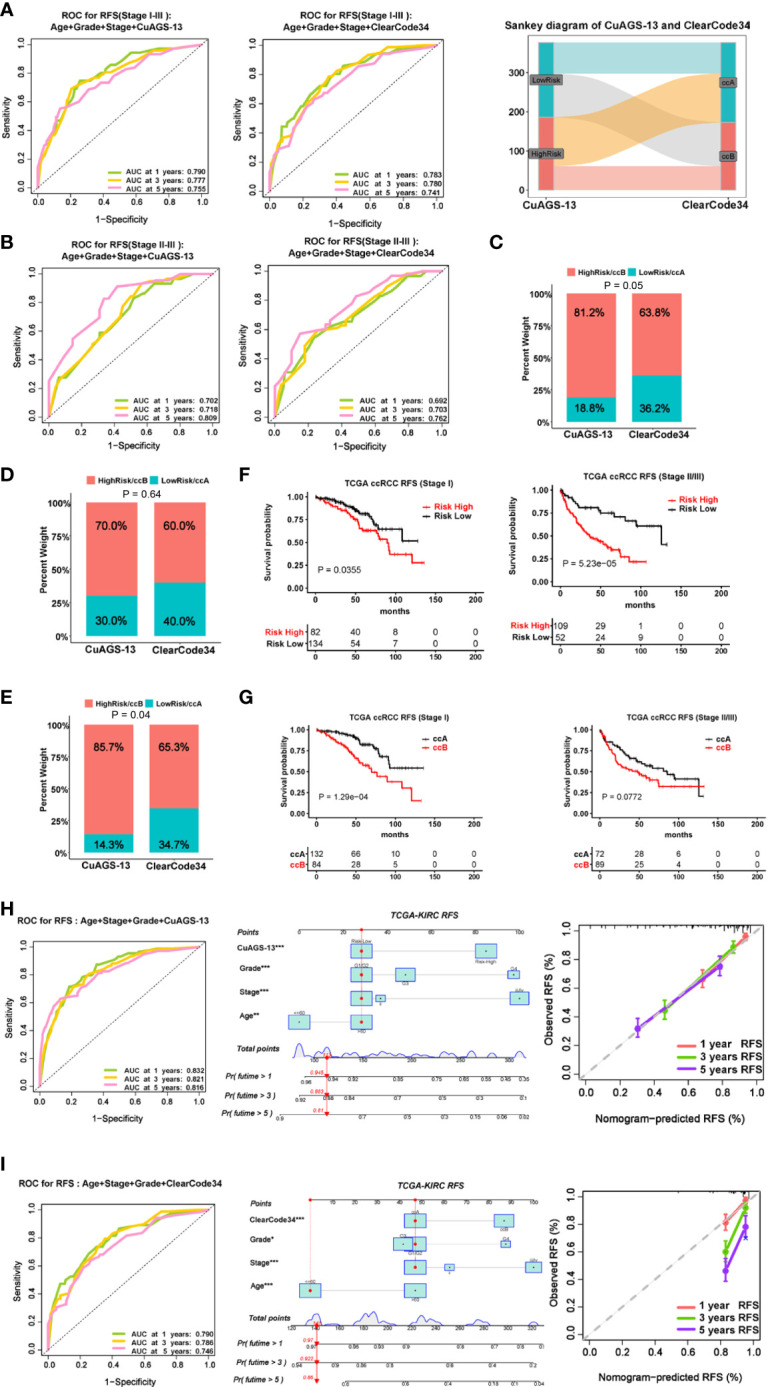
Comparison of predictive powers for recurrence and recurrence-free survival (RFS) between the CuAGS-13 and ccAccB/Clearcode34 models. **(A)** The ROC curve showing accuracy in predicting 1-, 3- and 5-year RFS for patients at stage I – III using CuAGS-13 (Left) and Clearcode34 (Middle) models. Right: The Sankey diagram showing different patient groups classified the CuAGS-13 and ccAccB/Clearcode34 models. **(B)** Left: The ROC curve showing accuracy in predicting 1-, 3- and 5-year RFS for patients at stage II – III using CuAGS-13 (Left) and ccAccB/Clearcode34 (Right) models. **(C)**: C-index analysis showing higher sensitivity of CuAGS-13 than Clearcode34 models for predicting recurrence in all patients at stage I – III. **(D)** C-index analysis showing no significant differences by CuAGS-13 and Clearcode34 models for predicting recurrence in patients at stage I **(E)** C-index analysis showing higher sensitivities of the CuAGS-13 than Clearcode34 models for predicting recurrence in stage II-III patients. **(F, G)** Kaplan–Meier survival analysis showing RFS predictive powers of CuAGS-13 **(F)** and Clearcode34 **(G)** models in patients at stage I and stage II – III, respectively. **(H)** The CuAGS-13 model-based nomogram for predicting 1-, 3- and 5-year RFS in TCGA ccRCC patients (stage I – IV). **(I)** The ccAccB/Clearcode34 model-based nomogram for predicting 1-, 3- and 5-year RFS in TCGA ccRCC patients (stage I – IV).

Approximately 30% of localized ccRCC (I – III stages) will relapse after surgery, and it is clinically important to stratify those patients with a higher recurrence risk. We thus assessed the value of the CuAGS-13 score in recurrence prediction. Because the ccA/ccB/ClearCode34 molecular classifier has been successfully applied for such a purpose, we also made a comparison between it and our CuAGS-13 score system. We first analyzed all the patients at I -III stages in the TCGA cohort. The time-dependent ROC curves showed comparable sensitivity and specificity for predicting recurrence-free survival (RFS) with both models when combined with age, stage and grade (**Figure 4A**). However, these two models classified different patient groups (<50% of overlapping) as revealed by the Sankey diagram (**Figure 4A** right panel). Because patients at stages II and III are more unpredictable, we further analyzed these patients separately. As shown in **Figure 4B**, the CuAGS-13 score performed better, in all three time points. Moreover, we employed multivariate and co-occurrence index (C-index) analysis to predict recurrence in 377 patients at I – III stages. Recurrence occurred in 69 patients with time information available, and obtained results demonstrated that 56/69 (81.2%) and 44/69 (63.8%) were in the CuAGS-13 high-risk group and ccB subtype, respectively (*P* = 0.05) (**Figure 4C**). Patients at stage I and II-III were then analyzed separately. Twenty of 216 stage I patients underwent recurrence, and the analysis results by these two models did not differ significantly (**Figure 4D**), while the CuAGS-13 score significantly outperformed the ccA/ccB/ClearCode34 model in predicting recurrence for patients at stage II and III (CuAGS-13 score *vs.* ccA/ccB: 85.7% *vs.* 65.3%, *P* = 0.04) (**Figure 4E**); A Kaplan-Meier analysis also showed a better stratification of RFS in the stage II – III patient group using the CuAGS-13 score, whereas the ccA/ccB/ClearCode34 model failed to predict RFS in this group (**Figures 4F, G**). Finally, we developed the CuAGS-13- and ccA/ccB/ClearCode34-based nomograms to predict RFS in the TCGA cohort of ccRCC (I – IV) (**Figures 4H, I**). The comparison of these two nomograms showed that the CuAGS-13 score-based nomogram exhibited a much higher consistence between predicted and observed recurrences (**Figures 4H, I**, left panels).

### The association between genomic alterations and the CuAGS-13 score in ccRCC

**Figure 5:**
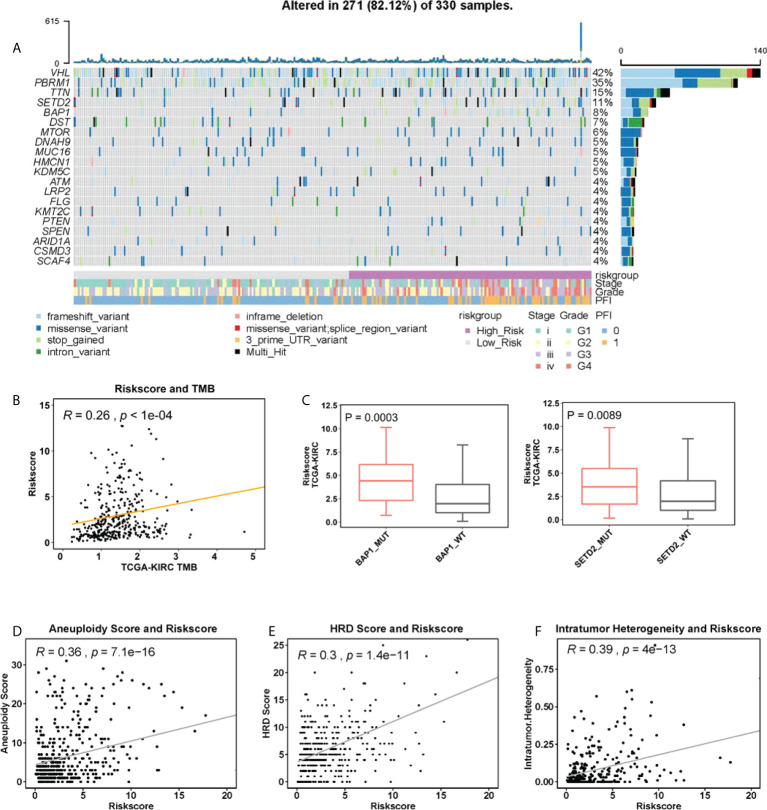
The association between genomic alterations and CuAGS-13 score in ccRCC. **(A)** The overview of the somatic mutations and relation to the CuAGS-13 score and clinical-pathological variables in the TCGA ccRCCs. **(B)** The positive correlation between CuAGS-13 score and tumor mutation burden (TMB) in the TCGA ccRCCs. **(C)** ccRCC tumors harboring BAP1 and SETD2 mutations exhibit significantly higher CuAGS-13 scores. **(D)** Positive correlation between the CuAGS-13 score and aneuploidy in ccRCC tumors. **(E)** Positive correlation between the CuAGS-13 score and homologous recombination deficiency (HRD) in ccRCC tumors. **(F)** Positive correlation between the CuAGS-13 score and intratumor heterogeneity in ccRCC tumors.

We next wanted to probe a potential link between the CuAGS-13 score and genomic alterations. Genomic data were available in 330 of 525 ccRCC tumors and 271 of them (81.12%) carried somatic mutations. The mutational landscape with 4% or more mutated genes was shown in **Figure 5A**. The following results were obtained from the analysis of ccRCC genomic alterations: (i) The CuAGS-13 score was significantly correlated with tumor mutation burden (TMB) in a positive manner (**Figure 5B**), and high-risk score tumors carried significantly a significantly higher frequency of BAP1 and SETD2 mutations (**Figure 5C**). (ii) The score and aneuploidy correlated positively with each other (**Figure 5D**). (iii) Homologous recombination deficiency (HRD) was highly correlated with the risk score (**Figure 5E**). In addition, intratumor heterogeneity (ITH), one of the key drivers for ccRCC evolution (38), was highly correlated with the CuAGS-13 score (**Figure 5F**).

### The enriched signaling pathways in ccRCC tumors with high CuAGS-13 score

We further sought to determine differences in signaling pathways between CuAGS-13 high and low risk tumors. In the TCGA cohort, the GSEA-KEGG and Hallmark analyses revealed 31 and seven pathways enriched in CuAGS-high tumors, respectively (**Figure S6**), and these pathways were mainly involved in metabolisms. Unexpectedly, the TCA cycle and oxidative phosphorylation were also significantly enriched in this group of tumors. The analysis of the E-MTAB-1980 cohort showed very similar results (**Figure S7**).

### The CuAGS-13 score as a predictor for response to ICI therapy or combination with Bevacizumab

**Figure 6:**
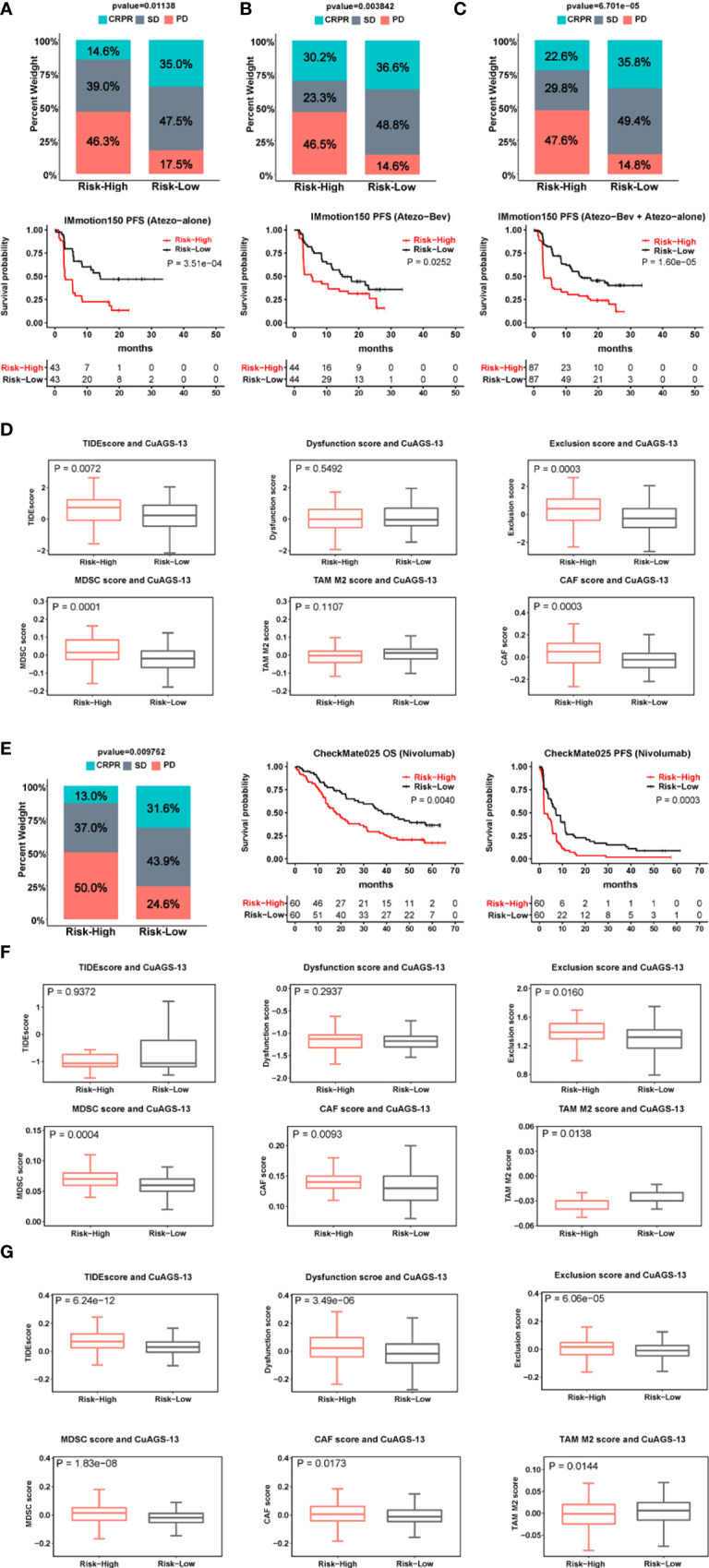
The CuAGS-13 score prediction of patient response to immune checkpoint inhibitors (ICIs) and combination with Bevacizumab in ccRCC. **(A–C)** The CuAGS-13 score prediction of patient response to Atezolizumab alone or Atezolizumab plus Bevacizumab in IMmotion150 trial. Differences in response rates and PFS between the CuAGS-13 high- and low-risk group patients treated with Atezolizumab alone **(A)**, Atezolizumab plus Bevacizumab **(B)** and all together **(C)**. **(D)** TIDE score analyses showing differences between the CuAGS-13 high- and low-risk group patients in IMmotion150 trial. **(E)** Differences in response rates and survival (OS and PFS) between the CuAGS-13 high- and low-risk group patients treated with Nivolumab in CheMate025 trial. **(F)** TIDE score analyses showing differences between the CuAGS-13 high- and low-risk group patients in CheMate025 trial. **(G)** TIDE score analyses showing differences between the CuAGS-13 high- and low-risk group patients in the TCGA ccRCC cohort.

ICI therapy has been applied to ccRCC patients, but there is still lack of established biomarkers reliably predicting response. We sought to evaluate whether the CuAGS-13 risk score could serve as such a predictor. The IMmotion150 phase II trial (34, 35), which included 263 ccRCC patients, was analyzed as the training cohort (**Table S4**). Among these patients, 86 received Atezolizumab therapy, 88 were treated with Atezolizumab in combination with Bevacizumab, and the rest 89 with Sunitinib. We first analyzed 86 patients treated with atezolizumab alone. Patient responses to Atezolizumab were divided into complete/partial remission (CRPR), stable disease (SD) and progressive disease (PD). CRPR, SD and PD in the high-risk group were 14.6%, 39% and 46.3%, respectively, while 35%, 47.5% and 17.5% in the low-risk group, respectively (*P* = 0.011) (**Figure 6A**). The median PFS for high- and low-risk groups were 3 and 14.1 months, respectively (*P* = 0.004, HR, 2.6 (1.61 – 4.65)) (**Figure 6A**). For 88 patients treated with both Atezolizumab and Bevacizumab, the CRPR rate increased robustly from 14.6% to 30.2% in the high-risk group patients, while was largely same in the low-risk group (35% *vs.* 36.6%) (**Figure 6B**). Nevertheless, patients with PD during the treatment were 3-time higher in the high- than low-risk groups (46.5% *vs.* 14.6%; *P* = 0.004) (**Figure 6B**); and the median PFS for high- and low-risk groups were 5.3 and 14.9 months, respectively (*P* = 0.025, HR, 1.8 (1.06 – 3.01)) (**Figure 6B**). Of note, in the CuAGS-13 high-risk group, the median PFS increased from 3.0 to 5.3 months when Bevacizumab was added, but this PFS increase was not statistically significant compared with that in patients treated with Atezolizumab alone (*P* = 0.20; HR, 1.36 (0.83 – 2.22)). Patients receiving Atezolizumab alone and plus Bevacizumab were then analyzed together, and increased CRPR while decreased SD rates were the major changes in the high-risk group compared with those in patients treated with Atezolizumab alone. The treatment results between high- and low-risk groups were CRPR, 22.6% and 35.8%; SD, 29.8% and 49.4%; PD, 47.6% and 14.8%, respectively (*P* = 6.7e-05) (**Figure 6C**). The median PFS for high- and low-risk group patients were 3.1 and 14.3 months, respectively (P = 1.6e-05; HR, 2.24 (1.52 – 3.29)) (**Figure 6C**). To probe how the CuAGS-13 score affects the efficacy of ICI therapy, we analyzed its relationship with Tumor Immune Dysfunction and Exclusion (TIDE) score, a computational framework to predict responses to immune checkpoint blockade and determine mechanisms underlying tumor immune escape (37). As shown in **Figure 6D**, the total TIDE score was significantly higher in the high-risk group. Consistently, exclusion, MDSC and CAF scores except M2 score were all higher in this group, whereas there were no differences in Dysfunction score between two groups (**Figure 6D**).

For validation, 120 ccRCC patients who received Nivolumab treatment in the CheckMate025 phase II trial (23, 24) were analyzed for their efficacy (**Table S5**). CRPR, SD and PD in the high-risk group were 13%, 37% and 50%, respectively, while 31.6%, 43.9% and 24.6% in the low-risk group, respectively (*P* = 0.01) (**Figure 6E**). The better efficacy in the low-risk group led to significantly longer patient OS and PFS (**Figure 6E**). The median PFS in the high- and low-risk groups was 2.1 and 7.2 months, respectively (*P* = 0.0003; HR, 1.98 (1.33 – 2.94) (**Figure 6E**), while OS was 17.9 and 38.4 months, respectively (*P* = 0.004; HR, 1.87 (1.21 – 2.89) (**Figure 6E**). These results were largely in accordance with those obtained from IMmotion150. There were no differences in the total TIDE score and T cell dysfunction score, however, T-cell exclusion, MDSC and CAF scores were significantly higher in the high-risk group (**Figure 6F**), which was consistent with the analysis result obtained from IMmotion150.

To further determine the relationship between the CuAGS-13 and TIDE scores, we analyzed the TCGA cohort of ccRCC. The total TIDE, dysfunction, exclusion, MDSC and CAF scores were all significantly higher, while TAM M2 score was lower in the high-risk group (**Figure 6G**). These findings favor an increased TIDE score in the CuAGS-13 high-risk group patients.

### The CuAGS-13 score as a predictor for response to Sunitinib treatment

**Figure 7:**
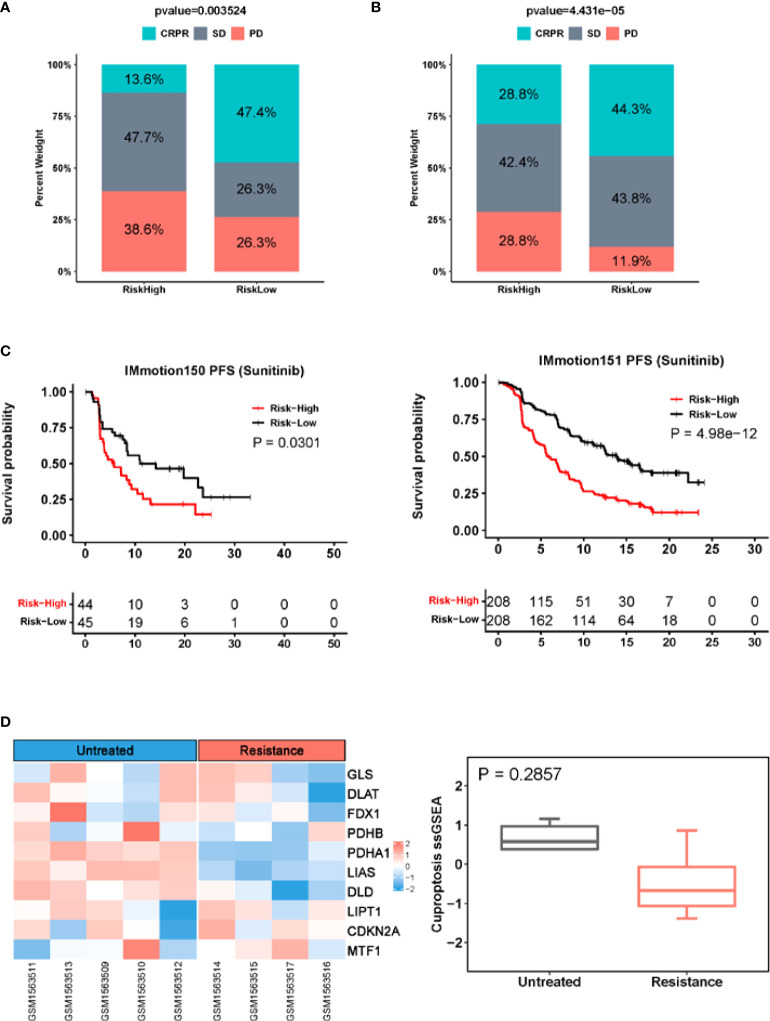
The CuAGS-13 score prediction of patient response to Sunitinib in ccRCC. **(A)** Differences in response rates between the CuAGS-13 high- and low-risk group patients treated with Sunitinib in IMmotion150 trial. **(B)** Differences in response rates between the CuAGS-13 high- and low-risk group patients treated with Sunitinib in IMmotion151 trial. **(C)** Significant association between shorter PFS and the CuAGS-13 high-risk group patients treated with Sunitinib in IMmotion150 trial (left) and IMmotion151 trial (right). **(D)** The lower cuproptosis score in Sunitinib-resistant PDX tumors. Microarray data in five untreated and four Sunitinib-resistant PDX tumors were analyzed for their cuproptosis score. Left panel: Heatmap showing expression of 10 cuproptosis factors. Right panel: The cuproptosis score in untreated and Sunitinib-resistant PDX tumors. A cuproptosis score was calculated using ssGSEA.

We also evaluated whether the CuAGS-13 score model could predict the efficacy in patients treated with Sunitinib. As documented above, Sunitinib was applied to 89 patients in the IMmotion150 cohort (34, 35), and the analysis results showed that the total CR and PR rate was more than 4-fold higher in the low- than high-risk groups (47.4% *vs.* 13.6%) (*P* = 0.004) (**Figure 7A**). The median PFS for high- and low-risk groups was 5.8 and 11 months, respectively (*P* = 0.03; HR, 1.77 (1.04 – 3.03) (**Figure 7C**). In the second cohort of 416 ccRCC patients treated with Sunitinib (IMmotion151) (18, 36) (**Table S6**), we obtained similar results (low- *vs.* high-risk: CRPR, 44.3% *vs.* 28.8%; SD, 43.8% *vs.* 42.4%; PD, 11.9 *vs.* 28.8%. *P* = 0.0004) (**Figure 7B**). In accordance with the findings above, patient PFS was significantly shorter in the high- than low-risk groups, and median PFS was 5.8 and 13.9 months, respectively (*P* = 5.0e-12; HR, 2.26 (1.78 – 2.88)) (**Figure 7C**). To determine whether Sunitinib affects the cuproptosis signaling, we analyzed the cuproptosis score in tumors derived from patient-derived xenografts (PDX) models in GSE64052 (39). Microarray data were available in five untreated and four Sunitinib-resistant PDX tumors and expression levels of 10 cuproptosis genes were listed in **Table S7**. As shown in **Figure 7D**, the Sunitinib-resistant tumors expressed lower cuproptosis scores than untreated ones, however, the difference was not statistically significant.

## Discussion

Approximately 30% of ccRCC patients with localized disease relapses after nephrectomy, and therefore stratifying recurrence risk is important, especially for patients at stage II and III whose clinical behaviors are precarious (2, 4). On the other hand, up to 30% ccRCC patients present metastasis at diagnosis and systemic treatments are required (2, 4). During the last decade, tyrosine-kinase inhibitors such as Sunitinib, VEGF antibody Bevacizumab and ICIs have been applied to metastatic or relapsed patients and good efficacy observed in a subset of ccRCCs (15, 17). Reliable biomarkers are required to accurately stratify recurrence risk, and to predict response to targeted therapeutic drugs and ICIs. In the present study, we addressed these issues by analyzing ccRCCs from the TCGA and other datasets to construct a cuproptosis-associated model for prediction of survival, recurrence and response to ICIs, Bevacizumab and Sunitinib.

Cuproptosis is copper-dependent cell death resulting from FDX1-mediated mitochondrial protein lipoylation. FDX1 reduces Cu^++^ to Cu^+^, while lipoic acid pathway effectors, especially lipoyl synthase (LIAS), together with FDX1, promote the lipoylation of the pyruvate dehydrogenase (PDH) complex-containing enzymes in the TCA cycle (32). The PDH complex includes dihydrolipoamide S-acetyltransferase (DLAT), pyruvate dehydrogenase E1 subunit alpha 1 (PDHA1), and pyruvate dehydrogenase E1 subunit beta (PDHB), and their lipoylation is required for enzymatic function (32). However, Cu^+^ directly binds to the lipoyl moiety in these lipoylated proteins, and if excessively accumulated, results in lipoylated protein aggregation, proteotoxic stress and eventual cell death (32). In addition, FDX1 and Cu^+^ facilitates degradation of Fe–S cluster proteins, which further enhances onsets of cuproptosis (32). It is currently unclear whether cuproptosis, like apoptosis or other types of RCD, has any roles in oncogenesis. Our analyses of the TCGA cohort of ccRCC showed that higher FDX1 expression is significantly associated with longer OS and PFS, and moreover, its downregulation occurs in ccRCC, which collectively indicates that cuproptosis may act as tumor suppressor in this cancer type. Moreover, according to correlation with cuproptosis factors, we identified a panel of cuproptosis-associated genes and developed the CuAGS-13 score model that could predict patient OS/PFS and recurrence risk with a high accuracy.

Gene expression patterns have been shown to improve cancer classification and prediction of patient outcomes, and several groups have developed expression profiling-based molecular tools for ccRCC prognostication (5–10, 40). For instance, Rini et al. introduced a 16-gene score for recurrence risk stratification in ccRCC patients at stage I - III (10), and Buttner et al. set up the S-3 score (the 97 gene signature based on gene expression in the terminal part of proximal tubules) for survival assessment (8). More recently, a 13-gene signature was constructed to predict risk and survival of ccRCCs. However, these expression signatures have not been well validated independently. There is another molecular classification score so-called ccA/ccB/ClearCode34 model (6, 7), which have been evaluated in several clinical observations, and consistently showed their robustness in outcome prediction of ccRCC (41). To test the stratification ability of the CuAGS-13 score, we further analyzed the same cohorts of ccRCCs from TCGA and E-MTAB-1980 using the ClearCode34 score and compared the predictive effectiveness as assessed by both models. The obtained results demonstrated that the CuAGS-13 score outperformed the ClearCode34 classifier in predicting recurrence risk and RFS. Further studies of additional cohorts of ccRCC are required to confirm the present findings.

Efforts have been made to search for predictors of ICI response in ccRCC patients, and several molecules are shown to be useful in some reports but fail to be validated by others (22–27, 42, 43). Intriguingly, the presence of high CD8 T cells in tumors is associated with poor prognosis (16). More recently, other biomarkers have been introduced to predict response to ICIs (28). Here we observed that the CuAGS-13 score assessment helped stratify ICI responders in two cohort patients who received either Atezolizumab or Nivolumab. In both cohorts, the CRPR rate was more than two-fold higher in the low- than high-risk group. Consistently, patients in the low-risk group had significantly longer PFS. The TIDE analysis revealed significantly higher T cell exclusion, MDSC and CAF scores while lower TAM M2 score in the high-risk group from both cohorts, which may contribute to poor response to ICIs in this group. It is currently unclear whether cancer immunotherapy is involved in cuproptosis. Recent studies showed that CD8 T cells promoted tumor cell lipid peroxidation and ferroptosis in patients treated with nivolumab (44, 45). It is thus worth probing the mechanistic relationship between cuproptosis and ICI efficacy, and if this is indeed the anti-tumor mechanism underlying ICI immunotherapy, targeting the cuproptosis pathway in combination with ICIs may be a novel therapeutic strategy.

Combination of anti-angiogenic therapy and ICIs has been shown to synergistically inhibit tumor growth and progression (19, 20). Targeting angiogenesis convert the tumor-immune environment from immune-suppressive to immune-supportive, thereby promoting the efficacy of ICIs. On the other hand, ICIs exert an anti-angiogenic effect (19, 20). However, it remains poorly defined which patients will benefit from this combination protocol (19). Interestingly, we observed that the combined treatment of Atezolizumab and Bevacizumab doubled a CRPR rate in CuAGS-13 high-risk group patients without improving the efficacy in low-risk group patients. Moreover, the increased CRPR seen in the high-risk group with the combined therapy was mainly derived from SD patients, because the PD rate was largely same between patients treated with Atezolizumab alone and Atezolizumab plus Bevacizumab. Based on the present findings, the Atezolizumab*/*Bevacizumab combination is suggested to apply to the CuAGS-13 high-risk patients.

Sunitinib has been widely used for ccRCC treatment (18, 36). We observed that patients who acquired CRPR were more than 3-time higher in the low-risk group compared with those in the high-risk group in IMmotion150 cohort treated with Sunitinib. The analysis of IMmotion151 cohort similarly showed significantly higher numbers of CRPR patients coupled with longer PFS in the low-risk group. These findings strongly suggest that the CuAGS-13 model can be used to predict Sunitinib responders in ccRCCs. It is currently unclear whether Sunitinib is associated with cuproptosis induction. Our preliminary results of GSE64052 PDX tumor analyses showed that Sunitinib-resistant tumors tended to have a diminished cuproptosis score, indicating possible escape of cuproptosis. Further cellular experiments and comparison of cuproptosis between Sunitinib-sensitive and resistant tumors are required to draw solid conclusions.

In our investigations, ccRCC tumors carrying BAP1 and SETD2 mutations exhibited higher CuAGS-13 scores. BAP1 is responsible for deubiquitinating H2K119, thereby impairing regulatory function of the polycomb repression complex1 (PRC1) in transcription, while SETD2 demethylate H3K36, leading to altered gene transcription (11, 38). During the ccRCC evolution, both BAP1 and SETD2 act as drivers for disease progression (38). It will be interesting to explore whether their mutations result in dysregulation of cuproptosis factors and escape of cuproptosis in ccRCCs. In addition, aneuploidy and HRD were significantly correlated with CuAGS-13 scores. Because BAP1 and SETD2 are required for genomic stability (38), their mutations may contribute to the correlation between them observed above. In addition, CuAGS-13 high-risk scores are significantly associated with male sex, senior age, higher grade tumors and advanced stages. Taken together, the CuAGS-13 model is a molecular classifier with many integrate features of ccRCC.

Metabolic reprogramming is a key feature of ccRCC due to the VHL inactivation and aberrant accumulation of HIF1/2α. Indeed, the GSEA analysis revealed that the enriched pathways were mainly involved in metabolic alterations in ccRCC tumors with CuAGS-13 high-scores, however, the enrichment of TCA and oxidative phosphorylation pathways were also observed in these tumors, which was unexpected. It was observed that SETD2 loss triggered a switch from glycolysis to OXPHS in ccRCC cells (46), while tumors with high CuAGS-13 score exhibited higher frequencies of SETD2 mutations, which might provide potential explanation. Likely, other unknown factors make contributions, too and further studies are required to elucidate this issue.

In summary, cuproptosis is the newly identified form of RCD, and based on its signaling molecules, we developed the CuAGS-13 score model that provides a robust tool to predict patient survival, recurrence, and response to ICIs, Bevacizumab and Sunitinib in ccRCC. This model, although derived from cuproptosis-related genes, is a classifier integrated with molecular and many other features of ccRCC. The present findings strongly suggest that the CuAGS-13 score system might significantly improve patient stratification for precision medicine of ccRCC, and it is worthy of validating these observations in clinical practices.

## Data availability statement

Source data downloaded from public databases are provided with this paper. Data from the IMmotion150 and151 trial were downloaded from European Genome-Phenome Archive (EGA) under accession number EGAS00001002928 and EGAC00001001813 with EGA approval. Any additional information required to reanalyze the data reported in this paper is available from the corresponding authors upon reasonable request.

## Author contributions

HY, YF, and DX conceived and designed the study. HY, JW, XQ, and QY performed bioinformatics analysis. YF and DX supervised the study. HY, YF, and DX wrote and revised the manuscript. All authors contributed to the article and approved the submitted version.

## Funding

This work was supported by grants from Scientific Research Foundation of Qilu Hospital of Shandong University (Qingdao) (No. QDKY2019QN17), National Natural Science Foundation of China (No. 82103557, and 81972475), Shandong Provincial Natural Science Foundation (No. ZR2020QH245), the Swedish Cancer Society No. 19 0018 Pj), Swedish Research Council (2018-02993), the Cancer Society in Stockholm (201393), and Karolinska Institutet (2018-01524).

## Conflict of interest

The authors declare that the research was conducted in the absence of any commercial or financial relationships that could be construed as a potential conflict of interest.

## Publisher’s note

All claims expressed in this article are solely those of the authors and do not necessarily represent those of their affiliated organizations, or those of the publisher, the editors and the reviewers. Any product that may be evaluated in this article, or claim that may be made by its manufacturer, is not guaranteed or endorsed by the publisher.
