# Supplementary material for: The cuproptosis-associated 13 gene signature as a robust predictor for outcome and response to immune- and targeted-therapies in clear cell renal cell carcinoma
Source: Front Immunol. 2022 Sep 5;13:971142. doi: 10.3389/fimmu.2022.971142 (PMC9483097; doi:10.3389/fimmu.2022.971142)
Supplement: Supplementary file 1 [file DataSheet_1.docx]

Supplementary Material

## Supplementary Figures (Figure S1 – S7)

**Supplementary Figure 1 (Figure S1)**. **Identification of the cuproptosis associated gene modules by the weighted gene co-expression network (WGCNA) analysis**. The analysis was performed using ‘WGCNA’ package and R software (version 4.0.2). (A) The sample clustering tree based on the WGCNA analysis of 525 ccRCC tumors and 72 non-cancerous renal tissues from the TCGA ccRCC cohort. Hierarchical clustering by average link shows no outliner samples. (B) Soft-thresholding value selection. Based on the scale-free fit index for various soft-thresholding powers (the scale independence, left panel) and mean connectivity for various soft-thresholding powers (mean connectivity, right panel), 6 was selected as a soft-threshold value (Scale-free R^2^  = 0.80). (C) The module eigengenes associated with the cuproptosis ssGSEA score. Using 6 as soft-thresholding, we constructed the adjacency matrix that was further transformed to the topological overlap matrix. A total of 27 modules were then identified, as shown in the heatmap.

**Figure S1**

**Supplementary Figure 2** (**Figure S2**). **The** **CuG-10 model and FDX1 expression for prediction of survival and response to ICI therapy in ccRCC patients**. (A) The schematic expression of CuG-10 model construction. The CuG-10 model was established directly based on expression of 10 cuproptosis genes (FDX1, LIAS, LIPT1, DLD, DLAT, PDHA1, PDHB, MTF1, GLS and CDKN2A) in the TCGA cohort of ccRCC tumors using ssGSEA analyses. (B) ROCs for CuG-10 in predicting 1, 3 and 5-year survival (OS and PFS) (TCGA cohort ccRCC). The median ssGSEA score value was used as the cutoff to divide patients into high- and low-risk CuG-10 groups. (C) ROCs for FDX1 expression in predicting 1, 3 and 5-year survival (OS and PFS) (TCGA cohort ccRCC). High- and low-FDX1 expression groups were categorized according to the median expression level. (D and E) Univariate and multivariate COX analyses of OS and PFS (TCGA cohort of ccRCC). In univariate COX analyses, the high-risk CuG-10 score predicts significantly shorter survival of both OS and PFS, while higher FDX1 expression was significantly associated with longer OS and PFS in the TCGA cohort of ccRCC patients. The multivariate analyses reveal that the CuG-10 model loses its prognostic power for both OS and PFS, whereas FDX1 expression is not an independent prognostic factor in patient PFS. (F to H) The comparison between ClearCode34 and CuG-10 scores for recurrence and RFS in the TCGA cohort of ccRCC. The ClearCode34 (F left panel) and CuG-10 (F right panel) exhibit largely same AUC areas in predicting recurrence at year 1, 3 and 5 in stage I – III patients (TCGA ccRCC). In univariate and multivariate analyses of RFS, the results from both models are very similar (G). Moreover, there is no significant difference in the accuracy of recurrence prediction between ClearCode34 and CuG-10 models (H). (I and J) No predictive impacts of the CuG-10 score (I) or FDX1 expression (J) on response to immune checkpoint inhibitor (ICI) and ICI + anti-angiogenic therapies in ccRCC patients (CheckMate025 and IMmotion151 cohorts). Atezo: Atezolizumab; Bev: Bevacizumab.

**Figure S2**

**Supplementary Figure 3 (Figure S3)**. **Comparisons among CuG-10, FDX1 and CuAGS-13 for prognosis of OS and PFS in ccRCC patients.** (A) The ROC curve showing accuracy in predicting 1-, 3- and 5-year OS and PFS for ccRCC patients (TCGA cohort) using CuG-10, FDX1 and CuAGS-13 models. The AUCs were < 0.70 in almost all CuG-10 and FDX1 predictions of OS and PFS (except CuG-10 for the 5-year PFS, 0.726), while between 0.70 and 0.82 for CuAGS-13, which demonstrated that the CuAGS-13 model performed much better. (B) The multivariate COX analyses revealing all of three models as independent prognostic factors for OS of ccRCC patients (TCGA cohort), however, the C-index was highest in CuAGS-13 while lowest in FDX1 (*P*<2.2e-16). (C) The multivariate COX analyses revealing all of three models as independent prognostic factors for PFS of ccRCC patients (TCGA cohort), however, the C-index was highest in CuAGS-13 while lowest in FDX1 (*P*<2.2e-16).

**Figure S3**

**Supplementary Figure 4 (Figure S4)**. Differences in protein expression of CuAGS-13 components between ccRCC tumors and normal renal tissues in the TCGA cohort. Expression data was obtained from Clinical Proteomic Tumor Analysis Consortium (<http://ualcan.path.uab.edu/index.html>). Protein expression data were available for 9 of 13 genes (TMEM214, CCM2, P3H4, FDX1, CDC42BPG, GNG7, WDR72, BSPRY and KDF1).

**Supplementary Figure 5 (Figure S5)**. Differences in mRNA expression of CuAGS-13 components between ccRCC tumors and normal renal tissues in the ICGC-RECA-EU cohort of ccRCC. The cohort includes 91 tumors and 45 normal renal samples. ****, ** and * indicate P < 0.0001, 0.01 and 0.05, respectively.


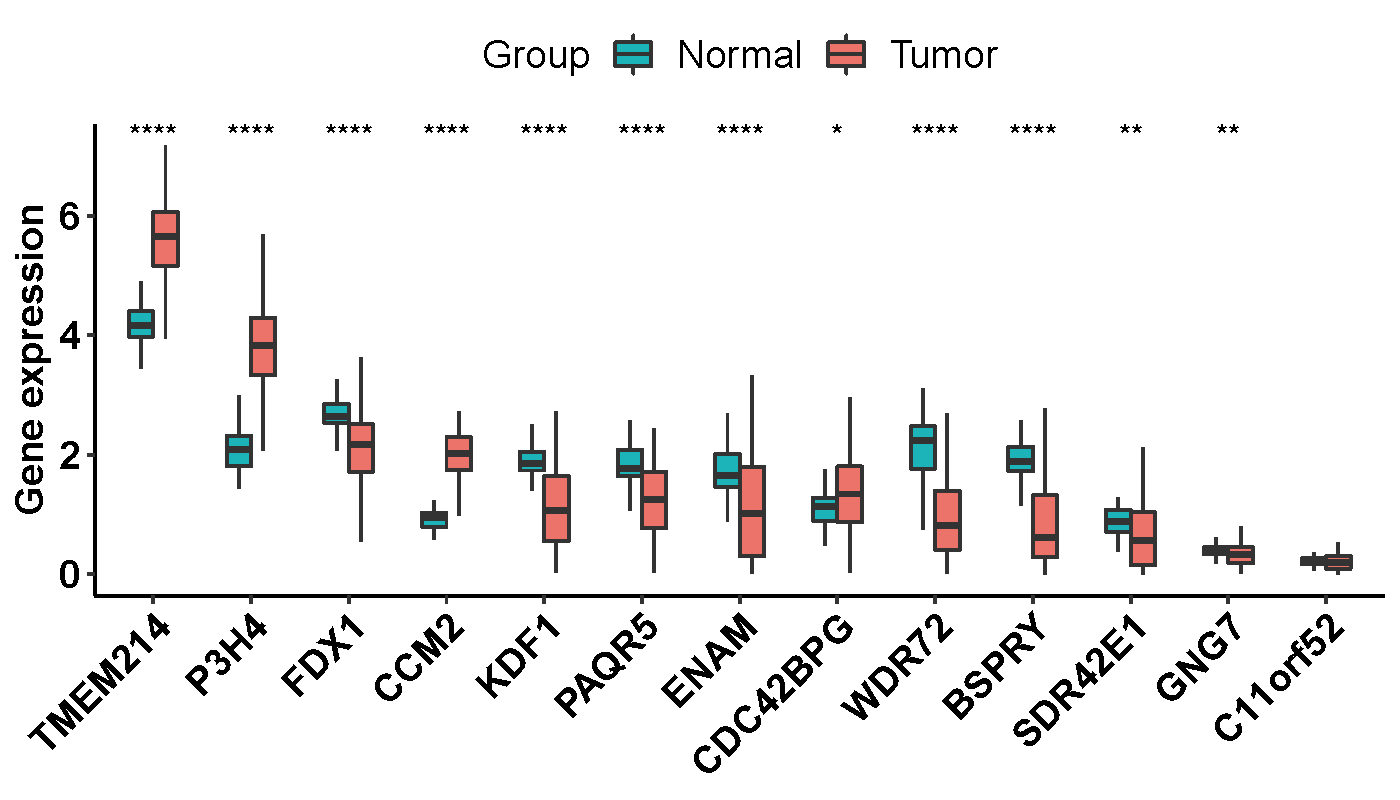


**Supplementary Figure 6 (Figure S6)**. Gene set enrichment analysis (GSEA) for KEGG (GSEA-KEGG) and Hallmark (GSEA-Hallmark) pathways (version 4.2.1 [www.broadinstitute.org/gsea](http://www.broadinstitute.org/gsea)) in the TCGA cohort of ccRCCs based on the CuAGS-13 ssGSEA score (High vs Low according to the median cut-off). The pathways enriched with *P* <0.05 and FDR < 0.25 are shown. (A) Left and right: The enriched GSEA-KEGG and GSEA-Hallmark pathways in CuAGS-13 high-tumors, respectively. Numbers in circles were affected genes in each signaling pathway. (B) Four enriched representative pathways in CuAGS-13-high tumors: pyruvate and fat acid metabolisms, TCA cycle and oxidative phosphorylation. NES, normalized enrichment score.

**Supplementary Figure 7 (Figure S7)**. Gene set enrichment analysis (GSEA) for KEGG (GSEA-KEGG) and Hallmark (GSEA-Hallmark) pathways (version 4.2.1 [www.broadinstitute.org/gsea](http://www.broadinstitute.org/gsea)) in the E-MTAB-1980 cohort of ccRCCs based on the CuAGS-13 ssGSEA score (High vs Low according to the median cut-off). The pathways enriched with *P* <0.05 and FDR < 0.25 are shown. (A) Left and right: The enriched GSEA-KEGG and GSEA-Hallmark pathways in CuAGS-13 high-tumors, respectively. Numbers in circles were affected genes in each signaling pathway. (B) Four enriched representative pathways in CuAGS-13-high tumors: pyruvate and fat acid metabolisms, TCA cycle and oxidative phosphorylation. NES, normalized enrichment score.

# Supplementary Tables (Table S1 – S7)

**Table S1. Clinic-pathological characteristics of the TCGA cohort of ccRCC patients and association with the CuAGS-13 score (High vs Low Risk)**

|  | TCGA-KIRC | |  |
| --- | --- | --- | --- |
|  | **Risk High**  *(n=262)* | **Risk Low**  *(n=263)* | **P value** |
| Age, mean (SEM^a^) | 62.0 (11.7) | 59.3 (12.5) | 0.009 |
| Sex, *n* (%) |  |  | 0.002 |
| Male | 188 (71.8) | 154 (58.6) |  |
| Female | 74 (28.2) | 109 (41.4) |  |
| Grade, *n* (%) |  |  | <0.001 |
| G1 | 1 (0.38) | 12 (4.67) |  |
| G2 | 86 (33.1) | 141 (54.9) |  |
| G3 | 108 (41.5) | 97 (37.7) |  |
| G4 | 65 (25.0) | 7 (2.72) |  |
| AJCC stage^b^, *n* (%) |  |  | <0.001 |
| I | 82 (31.4) | 181 (68.8) |  |
| II | 29 (11.1) | 28 (10.6) |  |
| III | 86 (33.0) | 38 (14.4) |  |
| IV | 64 (24.5) | 16 (6.08) |  |
| T, *n* (%) |  |  | <0.001 |
| T1 | 86 (32.8) | 183 (69.6) |  |
| T2 | 39 (14.9) | 30 (11.4) |  |
| T3 | 128 (48.9) | 49 (18.6) |  |
| T4 | 9 (3.44) | 1 (0.38) |  |
| Lymph node metastasis, *n* (%) |  |  | 0.012 |
| N0 | 118 (90.1) | 120 (98.4) |  |
| N1 | 13 (9.92) | 2 (1.64) |  |
| Metastasis, *n (%)* |  |  | <0.001 |
| M0 | 191 (76.4) | 230 (93.5) |  |
| M1 | 59 (23.6) | 16 (6.50) |  |
| White Cell, *n (%)* |  |  | 0.033 |
| Low | 5 (2.24) | 3 (1.44) |  |
| Normal | 147 (65.9) | 114 (54.8) |  |
| Elevated | 71 (31.8) | 91 (43.8) |  |

a, SEM, standard error of mean.

b,Tumor AJCC stages according to the American Joint Committee on Cancer (AJCC) 7th edition.

**Table S2. Clinic-pathological characteristics of the E-MTAB-1980 cohort of patients and association with the CuAGS-13 score (High vs Low Risk)**

|  | E-MTAB-1980 | |  |
| --- | --- | --- | --- |
|  | **Risk High**  *(n = 50)* | **Risk Low**  *(n = 51)* | **P value** |
| Age, mean (SEM^a^) | 64.4 (11.1) | 62.6 (11.9) | 0.436 |
| Sex, *n* (%) |  |  | 0.041 |
| Male | 43 (86.0) | 34 (66.7) |  |
| Female | 7 (14.0) | 17 (33.3) |  |
| Grade, *n* (%) |  |  | 0.006 |
| G1 | 7 (14.3) | 6 (12.0) |  |
| G2 | 22 (44.9) | 37 (74.0) |  |
| G3 | 15 (30.6) | 7 (14.0) |  |
| G4 | 5 (10.2) | 0 (0.00) |  |
| AJCC stage^b^, *n* (%) |  |  | 0.031 |
| I | 26 (52.0) | 40 (78.4) |  |
| II | 8 (16.0) | 2 (3.92) |  |
| III | 9 (18.0) | 4 (7.84) |  |
| IV | 7 (14.0) | 5 (9.80) |  |
| ClearCode34, *n (%)* |  |  | <0.001 |
| ccA | 20 (40.0) | 43 (84.3) |  |
| ccB | 30 (60.0) | 8 (15.7) |  |

a, SEM, standard error of mean.

b, Tumor AJCC stages according to the American Joint Committee on Cancer (AJCC) 7th edition

**Table S3. Clinic-pathological characteristics of the ICGC-RECA-EU cohort of patients and association with the CuAGS-13 score (High vs Low Risk)**

|  | ICGC-RECA-EU | |  |
| --- | --- | --- | --- |
|  | **Risk High**  *(n = 45)* | **Risk Low**  *(n = 46)* | **P value** |
| Age, mean (SEM^a^) | 60.1 (9.58) | 60.9 (10.5) | 0.705 |
| Sex, *n* (%) |  |  | 0.440 |
| Male | 28 (62.2) | 24 (52.2) |  |
| Female | 17 (37.8) | 22 (47.8) |  |
| Grade, *n* (%) |  |  | 0.191 |
| G1 | 7 (15.9) | 6 (13.0) |  |
| G2 | 19 (43.2) | 29 (63.0) |  |
| G3 | 8 (18.2) | 7 (15.2) |  |
| G4 | 10 (22.7) | 4 (8.70) |  |
| AJCC stage^b^, *n* (%) |  |  | 0.384 |
| I | 20 (48.8) | 28 (65.1) |  |
| II | 6 (14.6) | 6 (14.0) |  |
| III | 10 (24.4) | 5 (11.6) |  |
| IV | 5 (12.2) | 4 (9.30) |  |

a, SEM, standard error of mean.

b, Tumor AJCC stages according to the American Joint Committee on Cancer (AJCC) 7th edition

**Table S4. Characteristics of the IMmotion150 cohort of patients treated with Atezolizumab (Risk-High vs Low).**

|  | | IMmotion150 | | |  |
| --- | --- | --- | --- | --- | --- |
|  |  | **Risk High**  *(n = 87)* | | **Risk Low**  *(n = 87)* | **P value** |
| Stage, *n* (%) |  | |  | | . |
| IV | | 87 (100) | 87 (100) | |  |
| Metastasized, *n* (%) | |  |  | | . |
| Yes | | 87 (100) | 87 (100) | |  |
| Prior ICI^f^, *n* (%) | |  |  | | 0.145 |
| Tumorectomy | | 74 (85.1) | 81 (93.1) | |  |
| None | | 13 (14.9) | 6 (6.90) | |  |
| Treatment, *n* (%) | |  |  | | 0.879 |
| Atezo-alone | | 42 (48.3) | 44 (50.6) | |  |
| Atezo-Bev | | 45 (51.7) | 43 (49.4) | |  |
| ORR^e^, *n* (%) | |  |  | | <0.001 |
| CR | | 5 (5.95) | 11 (13.6) | |  |
| PR | | 14 (16.7) | 18 (22.2) | |  |
| SD | | 25 (29.8) | 40 (49.4) | |  |
| PD | | 40 (47.6) | 12 (14.8) | |  |

e, ORR, objective response rate:

CR, complete response;

PR, partial response;

SD, stable disease;

PD, progressive disease.

fICI, Imune checkpoint inhibitor.

**Table S5. Characteristics of the CheckMate025 cohort of patients and association with the CuAGS-13 score (Risk-High vs Low)**

|  | CheckMate025 | |  |
| --- | --- | --- | --- |
|  | **Risk High**  *(n = 60)* | **Risk Low**  *(n = 60)* | **P value** |
| Age, mean (SEM^a^) | 59.5 (13.1) | 62.4 (10.8) | 0.183 |
| Sex, *n* (%) |  |  | 0.015 |
| Male | 53 (88.3) | 41 (68.3) |  |
| Female | 7 (11.7) | 19 (31.7) |  |
| MSKCC^c^, *n* (%) |  |  | 0.005 |
| Favorable | 14 (23.3) | 22 (36.7) |  |
| Intermediate | 27 (45.0) | 33 (55.0) |  |
| Poor | 19 (31.7) | 5 (8.33) |  |
| IMDC^d^, *n* (%) |  |  | 0.001 |
| Favorable | 6 (10.0) | 14 (23.3) |  |
| Intermediate | 28 (46.7) | 39 (65.0) |  |
| Poor | 23 (38.3) | 6 (10.0) |  |
| Not reported | 3 (5.00) | 1 (1.67) |  |
| ORR^e^, *n* (%) |  |  | 0.015 |
| CRPR | 7 (11.7) | 18 (30.0) |  |
| SD | 20 (33.3) | 25 (41.7) |  |
| PD | 27 (45.0) | 14 (23.3) |  |
| NE | 6 (10.0) | 3 (5.00) |  |

a, SEM, standard error of mean.

c, MSKCC,Memorial Sloan Kettering Cancer Center(MSKCC) Prognostic Model,

d, IMDC, International Metastatic Renal Cell Carcinoma Database Consortium Criteria

e, ORR, objective response rate:

CR, complete response;

PR, partial response;

SD, stable disease;

PD, progressive disease;

NE, not estimated.

**Table S6 Characteristics of the IMmotion151 cohort of patients treated with Sunitinib and association with the CuAGS-13 score (Risk-High vs Low)**

|  | IMmotion151 | | |  |
| --- | --- | --- | --- | --- |
|  | **Risk High**  *(n = 208)* | **Risk Low**  *(n = 208)* | | **P value** |
| Age, mean (SEM^a^) | 59.3 (9.90) | | 60.2 (9.96) | 0.344 |
| Sex, *n* (%) | 87 (100) | | 87 (100) | 0.650 |
| Female | 54 (26.0) | | 49 (23.6) |  |
| Male | 154 (74.0) | | 159 (76.4) |  |
| Liver Metastases, *n* (%) |  | |  | 0.703 |
| no | 168 (80.8) | | 172 (82.7) |  |
| yes | 40 (19.2) | | 36 (17.3) |  |
| ORR^e^, *n* (%) |  | |  | <0.001 |
| CR | 2 (1.09) | | 7 (3.61) |  |
| PR | 51 (27.7) | | 79 (40.7) |  |
| SD | 53 (28.8) | | 23 (11.9) |  |
| PD | 78 (42.4) | | 85 (43.8) |  |
| a, SEM, standard error of mean  e, ORR, objective response rate.  CR, complete response;  PR, partial response;  SD, stable disease;  PD, progressive disease. |  | |  |  |
|  |  | |  |  |
|  |  | |  |  |
|  |  | |  |  |

| **Table S7. 10 cuproptosis gene expression in PDX tumors without treatment & Sunitinib resistance** | | | | | | | | | | |
| --- | --- | --- | --- | --- | --- | --- | --- | --- | --- | --- |
| Tumor | DLD | GLS | PDHB | PDHA1 | FDX1 | DLAT | MTF1 | LIPT1 | LIAS | CDKN2A |
| GSM1563509 (U) | 9,00 | 8,91 | 8,19 | 8,20 | 7,86 | 7,20 | 6,73 | 6,41 | 6,19 | 4,85 |
| GSM1563510 (U) | 9,05 | 8,78 | 8,37 | 8,17 | 7,83 | 7,13 | 6,95 | 6,31 | 6,19 | 4,63 |
| GSM1563511 (U) | 9,12 | 8,83 | 8,27 | 8,18 | 7,95 | 7,35 | 6,58 | 6,36 | 6,17 | 4,79 |
| GSM1563512 (U) | 9,08 | 9,07 | 8,18 | 8,22 | 7,97 | 7,35 | 6,64 | 6,1 | 6,17 | 4,00 |
| GSM1563513 (U) | 9,08 | 9,10 | 8,12 | 8,28 | 8,12 | 7,24 | 6,73 | 6,43 | 6,10 | 4,23 |
| GSM1563514 (SR) | 9,02 | 9,07 | 8,15 | 7,90 | 7,97 | 7,34 | 6,75 | 6,44 | 5,96 | 4,98 |
| GSM1563515 (SR) | 8,95 | 9,04 | 8,21 | 7,89 | 7,87 | 7,27 | 6,79 | 6,40 | 5,88 | 4,46 |
| GSM1563516 (SR) | 8,89 | 8,70 | 8,26 | 8,02 | 7,78 | 6,96 | 6,69 | 6,38 | 5,98 | 4,65 |
| GSM1563517 (SR) | 8,78 | 8,75 | 8,10 | 7,90 | 7,94 | 7,15 | 6,88 | 6,26 | 5,94 | 4,78 |
| U: Untreated;  SR: Sunitinib resistant |  |  |  |  |  |  |  |  |  |  |

mRNA level was expressed as log2(value+1) (based on the microarray probe values).
